# Soil fertility and rhizosphere microbiome affecting hydroxysafflor yellow A accumulation in safflower

**DOI:** 10.3389/fmicb.2025.1738669

**Published:** 2026-01-07

**Authors:** Wenjie Shen, Wanting Yang, Shuwei Qin, Yang Liu, Guojun Li, Xia Zhang, Mingqiang Bao, Yaqian Lu, Kexin Sun, Wei Ma, Hongbin Li, Asigul Ismayil, Aiping Cao

**Affiliations:** 1 Ministry of Education Key Laboratory of Xinjiang Phytomedicine Resource Utilization, Xinjiang Production and Construction Corps Key Laboratory of Oasis Town and Mountain-Basin System Ecology, College of Life Sciences, Shihezi University, Shihezi, China; 2 Agriculture, Forestry and Grassland Service Center, 15th Regiment, Tumshuk, Xinjiang, China; 3 Agricultural, Forestry, Grassland and Ecological Environmental Protection Center of Baiyang City, Xinjiang, China; 4 College of Life Sciences, Shihezi University, Shihezi, Xinjiang, China

**Keywords:** authentic medicinal materials, geographical environment, hydroxysafflor yellow A, plant-soil-microbe, safflower

## Abstract

**Introduction:**

Safflower (*Carthamus tinctorius* L.) is a prized medicinal species whose therapeutic value hinges on the abundance of bioactive metabolites. Accumulation of these metabolites are influenced by a range of environmental and edaphic factors, including soil physicochemical parameters, extracellular enzyme activities, composition and function of rhizosphere microbiome. However, how these factors individually and synergistically orchestrate the biosynthesis, transport, and ultimate storage of pharmaceutically active compounds within Safflower tissues remains unknown.

**Methods:**

Here, high-throughput amplicon sequencing coupled with comprehensive physiological profiling was employed to investigate soil characteristics, enzyme activities, and rhizosphere microbial communities of safflower across 36 soil samples collected at two distinct altitudes and two growth stages.

**Results:**

The effective component content was detected in 18 samples, and our results revealed that the safflower stigmas from the high- altitude site (YM) contained significantly elevated levels of hydroxysafflor yellow A (HSYA) compared to those from the lowland site (YF). Soils at the YM site exhibited markedly higher fertility, with available phosphorus, total nitrogen, and organic matter identified as key drivers of HSYA accumulation. Both sites showed high diversity and abundance in rhizosphere microbial communities, with Actinobacteria and Proteobacteria dominating the bacterial communities, and Ascomycota being the predominant fungal phylum.

**Discussion:**

Taken together, our findings show that soil properties, microbial communities, and climatic conditions work interactively to influence the buildup of bioactive compounds in safflower. These insights suggest that precise management of soil nutrients and the rhizosphere microbiome can improve medicinal safflower quality.

## 1 Introduction

Safflower (*Carthamus tinctorius* L.) is an annual herbaceous plant belonging to the genus *Carthamus* within the Asteraceae family. It exhibits drought resistance, cold tolerance, salt tolerance, and tolerance to poor soils, possessing significant economic, medicinal, and ecological value (Chen et al., 2022; Han et al., 2018; Hui-Zhen et al., 2015). With a long history of cultivation and medicinal use in China (Lifen et al., 2015; Xi-Qiao et al., 2015; Xueli et al., 2017; Zhao et al., 2007). Over 200 compounds have been isolated from safflower, with the core active ingredient hydroxy crocin (HSYA) being a unique flavonoid. It demonstrates significant therapeutic efficacy and widespread application in treating coronary heart disease, cerebral infarction, and antitumor conditions, while also holding substantial value in the food and cosmetics industries (Lakhdari et al., 2020; Huang et al., 2021). Furthermore, the active components in the filaments—hesperidin and hyperoside—exhibit potent antioxidant capabilities (Akyüz et al., 2021; Ali et al., 2016; Zhang M. et al., 2024; Zhu et al., 2017). Rutin improves microcirculation and reduces blood lipids (Ghorbani, 2017). Caffeic acid demonstrates outstanding anti-inflammatory and antibacterial properties (Gu et al., 2023; Mude et al., 2022). Safflower from Yumin County, Xinjiang, is widely recognized as a quintessential representative of authentic medicinal materials due to its high content of active compounds and significant medicinal value (Liu et al., 2020).

The authenticity of medicinal plants is profoundly influenced by the rhizosphere microenvironment, including soil physicochemical properties and microbial communities (Carrión et al., 2019; Kováčik and Klejdus, 2014). Soil factors and enzyme activity form the basis for regulating microbial structure: studies indicate that elevation gradients significantly affect soil total nitrogen, total phosphorus, organic carbon, and their ratios (Yashiro et al., 2018), while altering the activity of urease, sucrase, phosphatase, and other enzymes; soil pH and C/N ratio are dominant factors screening bacterial communities (Nielsen et al., 2010; Twongyirwe et al., 2013); altitude changes alter vegetation types and soil physicochemical properties through the combined effects of temperature and precipitation (Lu et al., 2013; Yashiro et al., 2018), thereby driving shifts in microbial community structure, with microbial diversity differences closely related to soil factors (Fierer and Jackson, 2006; George et al., 2021; Li et al., 2021; Shen et al., 2012). Microbes, serving as the plant’s “second genome,” enhance plant resistance to pests, drought, salinity, and heavy metals through organic matter decomposition, nutrient mineralization, and energy flow (de Zelicourt et al., 2013; Shukla et al., 2011; Singh et al., 2013; Tian et al., 2019; Wiesenbauer et al., 2025). Recent studies reveal that rhizosphere microbial interactions with citrus host immune systems activate terpenoid skeleton synthase expression, promoting monoterpene accumulation (Su et al., 2023); The microbial community structure in the rhizosphere of wild Atractylodes macrocephala differs significantly from that of cultivated varieties, with 16 bacterial genera and 10 fungal genera showing strong correlations with active components like polysaccharides and atractylone (Song et al., 2023). Plants dynamically regulate rhizosphere microbial community structure by secreting secondary metabolites such as flavonoids and alkaloids and coordinating innate immune responses, enabling precise recognition and enrichment of beneficial microorganisms (Chen et al., 2020; Kwak et al., 2018; Stringlis et al., 2018). Ultimately, soil-microbe interaction networks influence the accumulation of medicinal active compounds by affecting plant secondary metabolic processes (Chai et al., 2021; Wang et al., 2022; Wang et al., 2021).

This study employed high-throughput amplicon sequencing to investigate the effects of soil factors and rhizosphere microbial communities on the accumulation of active compounds in safflower from different altitudinal regions in Xinjiang (Yumin Mountainous Region YM and Yumin Plain YF). The findings partially elucidate the coupled relationship between active compound content, soil properties, and the rhizosphere microbiome.

## 2 Materials and methods

### 2.1 Study area and sampling sites

The study site is Yumin County, Xinjiang, a primary production area for safflower, located at 82°20′E, 45°45′N. This study selected two sites at different elevations: Yumin Mountains (YM) and Yumin Plain (YF). The average elevation of YM is 1,166 meters, while that of YF is 735 meters. Both areas have a history of continuous safflower cultivation exceeding 5 years, ensuring homogeneity in land use. Sampling employed a five-point sampling method, with soil collected from the safflower rhizosphere using the soil shaking technique. Sampling occurred in both YM and YF regions during the safflower vegetative stage (VR) and reproductive stage (RR). Three small plots (spaced 30–200 m apart) were established in each region for each growth stage. Within each subplot, three sampling points were randomly selected. At each point, five samples were collected using the S-shaped five-point method and pooled into a single composite sample. Thus, each group comprised nine independent biological replicates, yielding a total of 2 production areas × 2 growth stages × 9 biological replicates = 36 independent rhizosphere soil samples. After sampling, all plant residues and surface debris were removed. Soil samples were transported to the laboratory in sterile plastic bags packed with dry ice and processed within 24 h. Samples were sieved through a sieved through a 2 mm sieve and divided into three equal portions: one stored at −80 °C for DNA extraction and high-throughput sequencing; One portion was stored at 4 °C for determination of microbial functional activities; the third portion was air-dried for soil property determination.

The stigmas of safflower are specialized reproductive organs that have not yet differentiated during the vegetative growth stage. Therefore, all stigma samples were collected during the reproductive phase. Sampling design: In both the YM and YF production areas, three small plots (spaced 30–200 meters apart) were established. Within each small plot, three sampling points were randomly selected. At each point, five samples were collected using the S-shaped five-point method and pooled into a single composite sample. Thus, each group comprised 9 independent biological replicates, yielding a total of 2 production areas × 9 biological replicates = 18 independent samples.

### 2.2 HPLC determination of active ingredient content

Determined using the national standard HPLC method for safflower. Chromatographic conditions: Column: Agilent LC Column, 5 μm, 4.6 mm (ID) × 250 mm; Column temperature: 30 °C; Mobile phase: 0.4% phosphoric acid (D)-methanol (B), gradient elution (0–60 min, 95–5% D; 60–65 min, 5–5% D; 65–70 min, 5–95% D); Flow rate: 1 mL/min; Detection wavelengths: 403, 367 nm; Injection volume: 10 μL. Dissolve standards in methanol for later use. Sample extraction: Accurately weigh 0.3 g dried safflower stigmas powder (passed through 80-mesh sieve), add 30 mL 25% methanol, extract using ultrasonication for 40 min, filter through 0.22 μm organic membrane for analysis.

### 2.3 Determination of soil physicochemical properties and enzyme activity

Air-dried soil samples were analyzed for the following properties: pH, electrical conductivity, moisture content, total potassium, total nitrogen, total phosphorus, alkali-hydrolyzable nitrogen, available potassium, available phosphorus, and organic matter. Following the soil physicochemical characteristics outlined in Bao Shidan’s *Soil Agrochemical Analysis*, an OHHUS for pH and conductivity measurements, semi-micro Kjeldahl method for total N, flame photometer for total K, HClO₄-H₂SO₄ digestion for total P, an alkaline diffusion method for alkali-hydrolyzable nitrogen, NH₄OAc extraction–molybdenum blue colorimetry for available P, and an external calorimeter for organic matter. Determination of soil urease, neutral phosphatase, soil sucrase, soil catalase, and soil cellulase was performed using kits produced by Solarbio Technology Co., Ltd., following the specific procedures outlined in the kit manuals.

### 2.4 Genomic DNA extraction and amplified fragment sequencing

Genomic DNA was extracted using the cetyltrimethylammonium bromide (CTAB) method. Soil community DNA was extracted from soil samples (0.5 g) using the FastDNA® SPIN Soil Kit (MP Biomedicals, California, USA), then stored at −20 °C and permanently preserved at −80 °C. The quantity and quality of isolated DNA were assessed using a NanoDrop spectrophotometer (Thermo Fisher Scientific ND-1000, Waltham, MA, USA) and 2.0% agarose gel electrophoresis (Bio-Rad, Hercules, CA, USA), respectively. The V4 hypervariable regions of the bac-terial 16S rRNA gene and internal transcribed spacer 1 (ITS1) regions of the fungal 18S rRNA gene were selected for sequencing analysis by using the universal primers 515\u00B0F (5′-GTGCCAGCMGCCGCGGTAA-3′) and 806R (5′-GGACTACNVGGGTWTCTAA-3′) for V4 region amplification, and the universal primers ITS5-1737F (5′-GGAAGTAAAAGTCGTAACAAGG-3′) and ITS-2043R (5′-GCTGCGTTCTTCATCGATGC-3′) for ITS1, respectively, with the forward primers modified to contain a unique barcode. PCR amplification steps were as follows: 95 °C pre-denaturation for 3 min; 30 cycles (95 °C denaturation for 30 s, 53 °C annealing for 30 s, 72 °C extension for 45 s); followed by a final extension at 72 °C for 10 min. PCR reactions were performed in 20 μL reaction mixtures with triplicate replicates, comprising: 10 μL 2 × Taq PCR master mix, 2 μL DNA template (10 ng), 0.8 μL each primer (10 μM), 0.2 μL BSA, and 6.4 μL sterile water, totaling 20 μL. PCR products were pooled separately, and 2 μL subsamples from each product were quantified via 2% agarose gel electrophoresis. All samples underwent three rounds of amplification, with the target PCR product fragments of expected size pooled into a single pool. This pool was subsequently visualized on a 2% agarose gel and further purified using a gel extraction kit (Qiagen). The TruSeq™ DNA Sample Kit was used. The amplicon library was constructed according to the standard protocol provided by Illumina, Inc. (San Diego, CA, USA). Quantification was subsequently performed using a Qubit Fluorometer (Invitrogen, Life Technologies, Grand Island, NY, USA) and a C1000 Real-Time Quantitative PCR (qPCR) System (Bio-Rad™, USA) equipped with a CFX96 thermal cycler, employing the SYBR Premix Ex Taq Real-Time Quantification Kit (per manufacturer’s instructions). Amplicon libraries with distinct index labels were pooled and sequenced using Illumina HiSeq 2,500 high-throughput sequencers for 2 × 250 bp paired-end sequencing. Sequencing data have been submitted to the SRA database (accession: PRJNA604670).

### 2.5 Data analysis

All statistical analyses were performed using R software version 4.3.0 (R Core Team, 2023) and SPSS, employing packages including vegan 2.6–4, ggplot2 3.4.2, and plspm 0.5.1. The significance level was uniformly set at *p* < 0.05. Prior to applying parametric tests, normality was assessed using the Shapiro–Wilk test and homogeneity of variance was evaluated using Levene’s test. Principal Component Analysis (PCA) was employed to visualize significant effects across treatments. Two-way ANOVA was used to test the interaction effects between region (YM/YF) and growth stage (VR/RR), with Tukey HSD post-hoc comparisons. Mantel tests assessed correlations between microbial communities, soil enzyme activities, and soil physicochemical properties with safflower bioactive compounds. Redundancy analysis (RDA) quantified the direct influence of soil physicochemical properties on bioactive compounds. Partial Least Squares Path Analysis (PLS-PM) was employed to explore the associative structure among soil, microorganisms, and metabolites. Model goodness-of-fit was assessed using the Goodness-of-Fit (GoF) index and R^2^. Path coefficient significance was determined through 500 bootstrap tests.

## 3 Results

### 3.1 Principal component analysis of YM and YF from different origins

**Figure 1:**
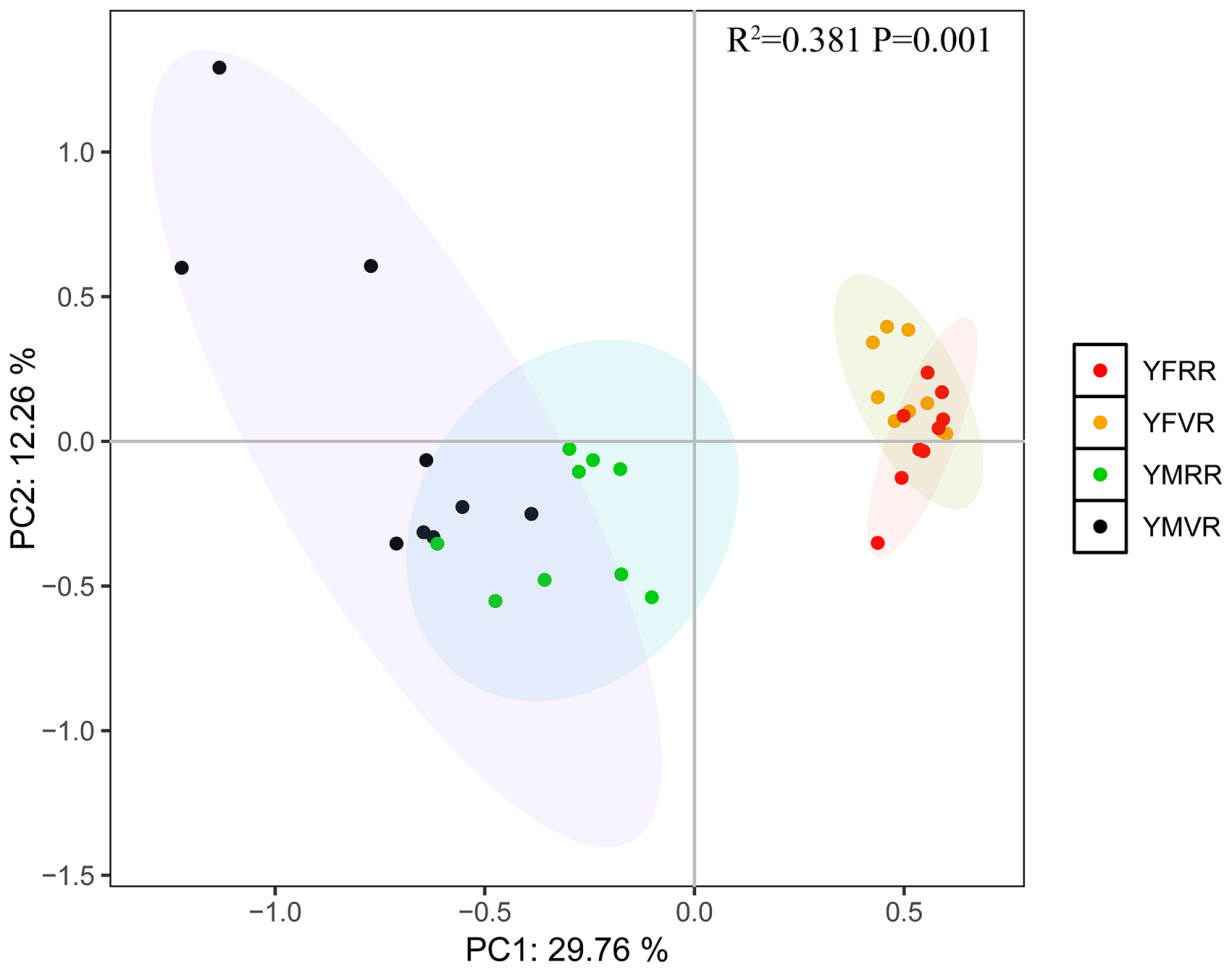
Principal component analysis of active compounds, soil physicochemical properties, soil enzyme activity, and soil rhizosphere microbiome for YFRR, YFVR, YMRR, and YMVR. All data represent biological replicates (*n* = 9 independent samples per group, total *n* = 36).

Principal Component Analysis (PCA) of safflower soil physicochemical properties, soil enzyme activity, soil rhizosphere microbiome, and active compounds in safflower stigmas across varying altitudes and developmental stages revealed distinct clusters in the PCA plot. YM and YF samples separated along the PC1 axis, with the first two principal components (PC1 and PC2) explaining most of the variance: PC1 accounted for 29.76% and PC2 accounted for 12.26% (Figure 1). PCA analysis indicates that altitude explains 29.76% of the variation in soil physicochemical properties, enzyme activity, and microbiome composition, suggesting altitude is a significant environmental variable.

### 3.2 Comparative analysis of active compounds in safflower stigmas from different origins

**Figure 2:**
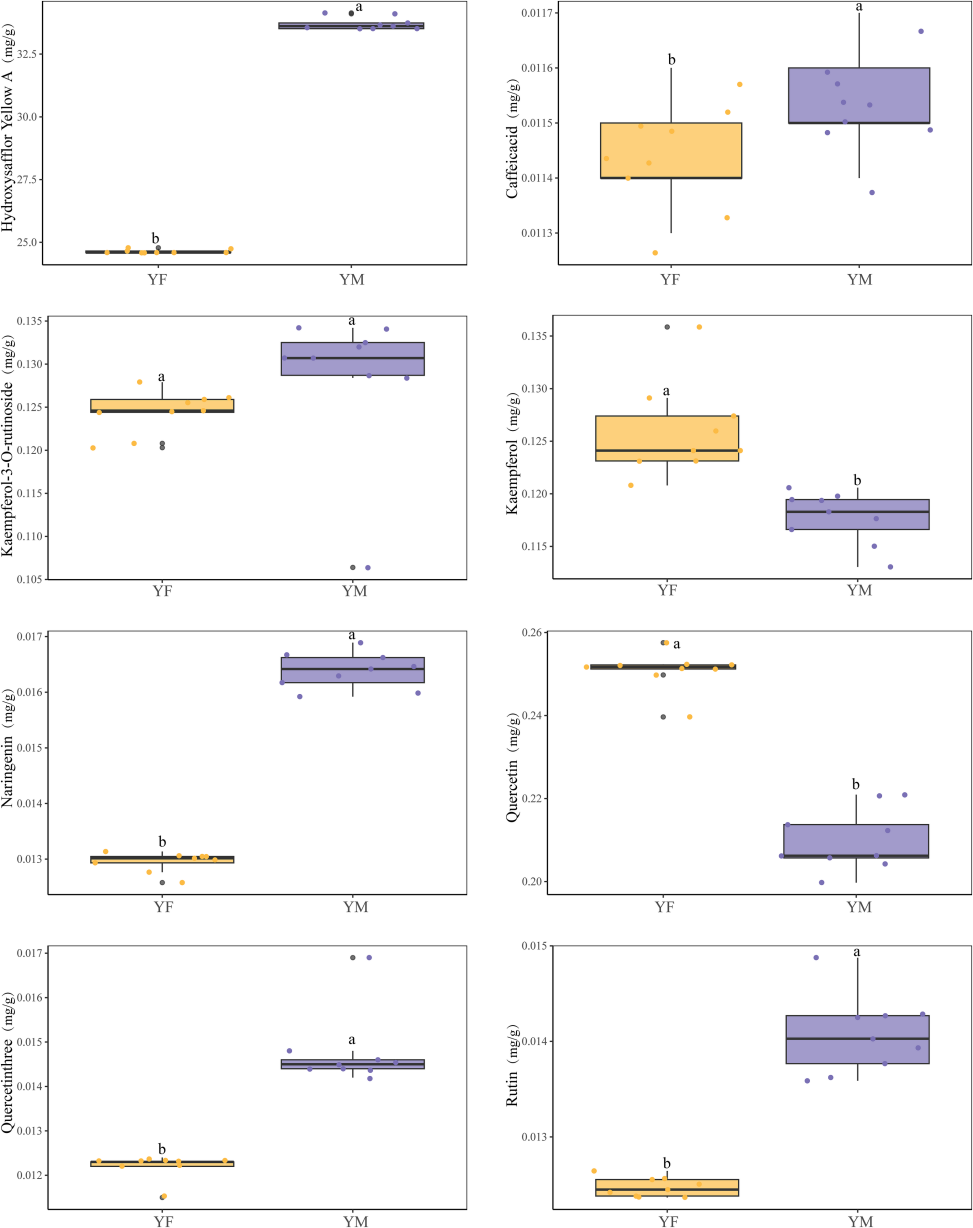
Box plot showing active compound content between YM and YF. Different letters indicate significant differences between groups (*p* < 0.05, Student’s *t*-test). Data are presented as biological replicates for reproductive stage only (*n* = 9 independent samples per group, total *n* = 18).

Active compounds in safflower stigmas were analyzed using HPLC methods. Since stigmas are present only during the reproductive phase of safflower, subsequent analyses of active components included only data from the reproductive phase, with no data from the vegetative phase incorporated into the analysis. Results indicate that 42 compounds were detected in safflower stigmas. Among these, eight active components with relatively higher concentrations in YM and YF were compared. The stigma-specific compound HSYA exhibited significantly higher content in YM than in YF, with YM levels reaching 1.36 times those of YF (Figure 2). Additionally, higher concentrations of naringenin, rutin, hyperoside, and caffeic acid were observed in YM-produced safflower stigmas (Figure 2). The results indicate that different altitudes influence the formation of active components in safflower stigmas, with the high-altitude YM region being more conducive to the accumulation of these compounds.

### 3.3 Comparative analysis of soil physicochemical properties and enzyme activities across origins and their correlation with key active components in safflower stigmas

**Figure 3:**
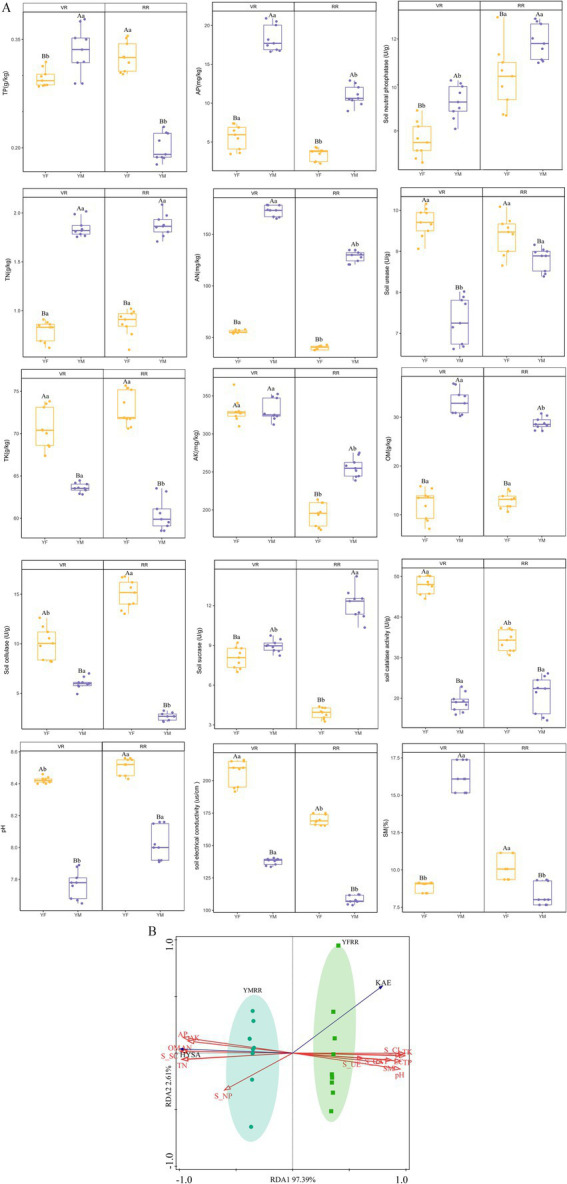
Comparison of physicochemical properties and enzyme activities among soils from different origins. **(A)** Soil characteristics of the YM sample differed from those of the YF sample. Different letters indicate significant differences (*p* < 0.05, two-way ANOVA followed by Tukey’s HSD test). All data represent biological replicates (*n* = 9 independent samples per group, total *n* = 36). Soil total nitrogen (TN, g kg^−1^); soil total phosphorus (TP, g kg^−1^); soil total potassium (TK, g kg^−1^); soil pH; soil ammonium nitrogen (AN, mg kg^−1^); soil available phosphorus (AP, mg kg^−1^); soil available potassium (AK, mg kg^−1^); soil organic matter (OM, g kg^−1^); soil electrical conductivity (EC, μS cm^−1^); soil moisture content (SM, %); soil catalase activity (S-CAT, U g^−1^); soil cellulase (S-CL, U g^−1^); soil neutral phosphatase (S-NP, U g^−1^); soil sucrase (S-SC, U g^−1^); soil urease (S-UE, U g^−1^). All soil enzyme activity units are expressed as U/g, μmol substrate per minute per gram dry soil. **(B)** Redundancy analysis of soil physicochemical properties and available nutrients; data are presented as biological replicates for reproductive stage only (*n* = 9 independent samples per group, total *n* = 18).

Soil physicochemical properties serve as key indicators of soil fertility. Ten physicochemical factors and five soil enzyme activities were measured in rhizosphere soils from two origins during the vegetative and reproductive stages of safflower. Comparative analysis revealed distinct differences in soil physicochemical properties between YM and YF. First, YM exhibited higher levels of TN, AN, AP, AK, OM, and SM compared to YF (Figure 3A). Specifically, AP and AN concentrations in YM were 5.22- and 3.26-fold higher than the YF average, respectively (Figure 3A). Second, during the VR stage, AP, AN and OM levels in YM exceeded those in YF by 3.42-, 3.13- and 2.51-fold, respectively (Figure 3A). During the RR stage, YM’s AN, AP, and OM contents were 3.32-, 3.06- and 2.25-fold higher than YF (Figure 3A). Overall, YM soil exhibited significantly superior comprehensive fertility compared to YF. Throughout safflower’s growth stages, YM soil maintained a pronounced advantage in nutrient content, particularly for key physicochemical factors like phosphorus, nitrogen, and organic matter. VR stage showed significantly higher AP and AK content than RR stage, while TN and pH were higher in RR than VR. This indicates greater demand for phosphorus and potassium during the VR stage, with increased nitrogen requirements in the RR stage. The TK, EC, and pH values in the YF region were all higher than those in the YM region, averaging approximately 1.22 -fold higher (Figure 3A). Soil enzymes are biologically active substances with catalytic functions secreted by microorganisms, plant roots, and animals in the soil. They play an active role in the conversion of soil matter and energy, serving as an active reservoir for plant nutrition. Their activity is closely linked to the state of the land (Wang et al., 2017; Xiaobing et al., 2002). A comparative analysis of soil enzyme activity at different developmental stages of safflower from two production areas reveals the following: First, YM showed 1.8-fold greater S-SC activity than YF, whereas YF exhibited 2.39-, 2.60-, 1.25- and 1.01-fold higher S-CAT, S-CL, S-UE and S-NP activities, respectively (Figure 3A). Second, during the VR stage, S-SC and S-NP activities were 1.07- and 1.03-fold higher in YM than in YF, while YF’s S-CAT, S-CL, and S-UE activities were 2.20-, 1.65- and1.32-fold higher than YM’s (Figure 3A). During the RR stage, YM exhibited S-SC and S-NP activities 3.46-and 1.09-fold higher than YF, respectively, while YF demonstrated S-CL, S-CAT, and S-UE activities 5.10-, 1.76- and 1.09-fold higher than YM, respectively (Figure 3A).

Redundancy analysis results reveal the relationship between soil properties and the primary active components HSYA and KAE in safflower stigmas. The active component HSYA showed positive correlations with AP, AN, OM, TN, and AK variables, and negative correlations with TK, TP, SM, EC, S-CL, S-UE, S-CAT, and pH variables. The active component KAE exhibited a negative correlation with S_NP (Figure 3B). In summary, soil properties are closely associated with the accumulation of active components in safflower stigmas. Physicochemical factors positively correlated with HSYA and KAE may represent key soil factors influencing these active components.

### 3.4 Microbial composition and Indicator groups from different origins

**Figure 4:**
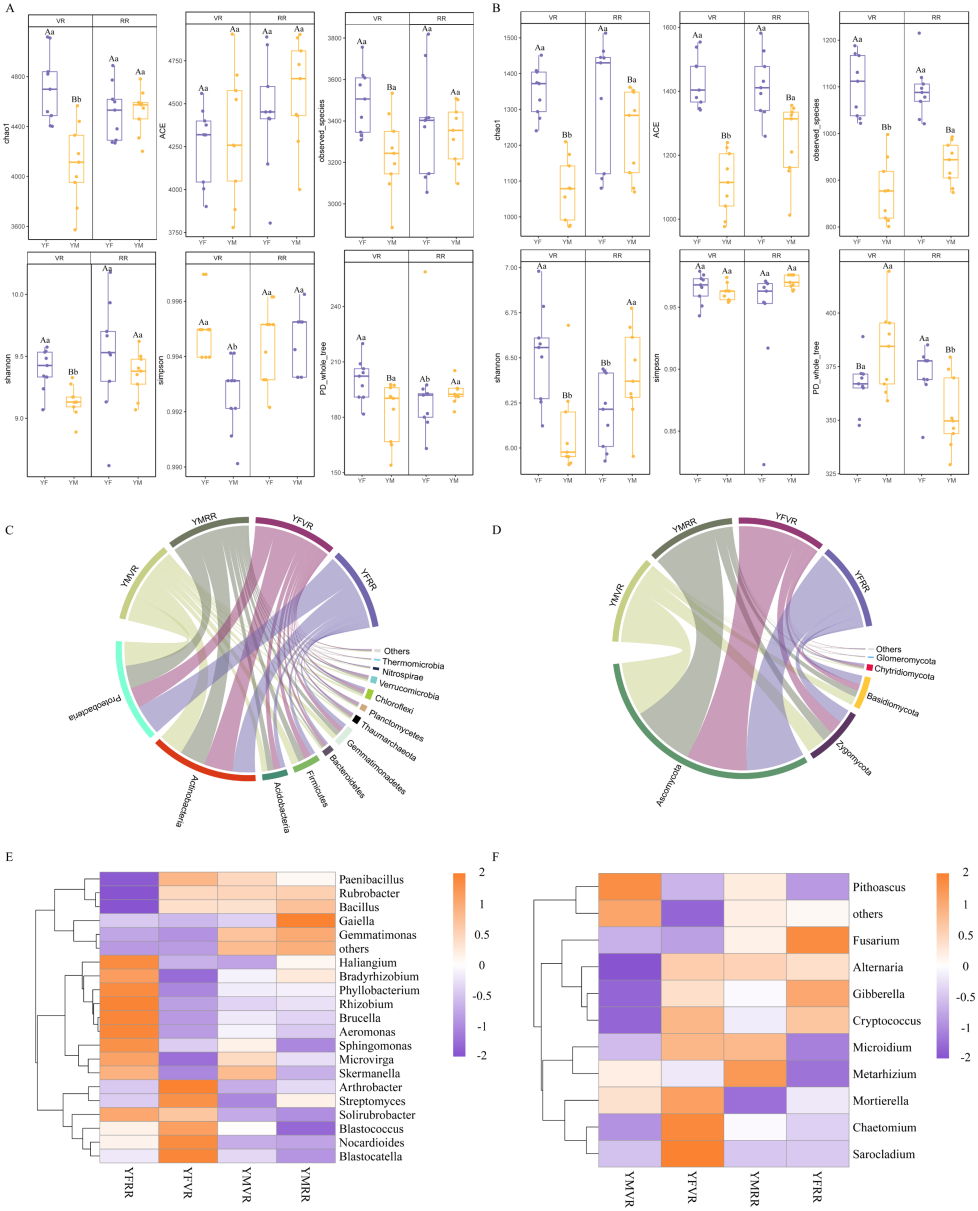
Effects of YM and YF on soil rhizosphere microbial communities. All data represent biological replicates (*n* = 9 independent samples per group, total *n* = 36). Different letters indicate significant differences (*p* < 0.05, two-way ANOVA followed by Tukey’s HSD test). **(A)** Bacterial diversity. ACE index; Observed Species; Shannon index; Simpson index; Chao1 index; PD-whole-tree (phylogenetic diversity). **(B)** Fungal diversity. ACE index; Observed Species; Shannon index; Simpson index; Chao1 index; PD-whole-tree (phylogenetic diversity). **(C)** Bacterial phylum classification diagram. **(D)** Fungal phylum classification diagram. **(E)** Bacterial genus heatmap. **(F)** Fungal genus heatmap.

Analysis of microbial community structure and abundance values in rhizosphere soils of safflower from different origins at various growth stages. Diversity indices for bacteria and fungi in different soils, as shown in Figure 4, indicate bacterial coverage exceeding 99.4% and fungal coverage exceeding 96.8% for each sample; The Chao 1 richness indices for bacteria and fungi ranged from 4,100 to 4,700 and 1,090 to 1,420, respectively, while ACE indices ranged from 4,260 to 4,625 and 1,150 to 1,410, respectively; Microbial diversity showed positive correlations with Shannon and Simpson indices. Bacterial and fungal Shannon indices ranged from 9.1 to 9.6 and 5.95 to 6.58, respectively, while Simpson indices ranged from 0.992 to 0.995 and 0.96 to 0.98, respectively (Figures 4A,B). This indicates that the rhizosphere soils of the two safflower plots harbor exceptionally rich microbial species, capturing most of the microbial taxa present.

At the phylum level, differences in relative abundance of bacteria and fungi were observed across soil samples. Actinobacteria and Proteobacteria constituted the dominant bacterial communities in safflower rhizosphere soils, accounting for 29.07–31.61% and 25.64–38.90%, respectively. Except for YFRR, Actinobacteria dominated followed by Proteobacteria followed by Firmicutes (6.60–9.67%), Acidobacteria (6.63–8.35%), Gemmatimonadetes (5.56–6.35%), Bacteroidetes (2.64–3.93%), Chloroflexi (2.78–3.20%), Thaumarchaeota (2.10–2.43%), Verrucomicrobia (1.31–2.62%), and Planctomycetes (1.45–2.46%) (Figure 4C). The phyla Firmicutes and Gemmatimonadetes exhibited higher proportions in YMVR, while Gemmatimonadetes, Thaumarchaeota, and Verrucomicrobia were more abundant in YMRR. Actinobacteria, Acidobacteria, Bacteroidetes, Chloroflexi, and Planctomycetes dominated YFVR, and the phylum Proteobacteria was predominant in YFRR. In the fungal community composition of safflower rhizosphere soil, Ascomycota (64.76–76.75%) dominated, followed by Zygomycota (12.74–22.50%), Basidiomycota (7.73–10.64%), Chytridiomycota (1.24–2.05%), and Glomeromycota (0.27–0.38%) (Figure 4D). The phyla Zygomycota, Basidiomycota, and Glomeromycota exhibited higher proportions in YMVR, while Ascomycota dominated in YMRR and Chytridiomycota in YFVR. Results indicate that bacterial community composition at the phylum level was similar between YM and YF, whereas relative abundances of most fungal groups were significantly higher in YM than in YF.

At the genus level, Rubrobacter (6.75–11.98%) and Arthrobacter (6.55–12.27%) were the dominant bacteria in the red clover rhizosphere soil, followed by Sphingomonas (3.44–6.32%), Blastococcus (3.19–3.52%), Solirubrobacter (2.65–3.38%), Gaiella (2.85–3.08%), Skermanella (2.22–2.63%), Streptomyces (2.02–2.89%), Microvirga (2.07–2.49%), and Bacillus (1.99–2.39%) (Figure 4E). Among these, Rubrobacter, Gaiella, and Bacillus exhibited higher abundances in YMRR, while Arthrobacter, Blastococcus, and Streptomyces showed higher abundances in YFVR. Sphingomonas, Solirubrobacter, Microvirga, and Skermanella were more abundant in YFRR. Gibberella (9.19–21.95%) and Mortierella (7.80–18.60%) were the dominant fungal genera in safflower rhizosphere soil, followed by Alternaria (1.03–11.85%), Fusarium (4.30–647%), Microidium (1.96–5.85%), Cryptococcus (2.09–5.14%), Chaetomium (2.48–5.64%), Metarhizium (2.57–4.80%), Pithoascus (0.24–8.63%), and Sarocladium (0.06–12.33%) (Figure 4F). Among these, Pithoascus was more abundant in YMVR, Metarhizium was more abundant in YMRR, while Mortierella, Alternaria, Microidium, Chaetomium, Cryptococcus, and Sarocladium were more abundant in YFVR. Gibberella and Fusarium were more abundant in YFRR. Soil rhizosphere microbial genus-level studies in safflower revealed that the abundance of the bacterial genus Rubrobacter averaged 1.29 times higher in YM than in YF. Similarly, the fungal genera Metarhizium and Pithoascus exhibited significantly higher abundances in YM than in YF. However, in YM medium, the genera Rubrobacter, the fungal genus Metarhizium, and the genus Pithoascus exhibited higher abundance. It is speculated that these microbial groups may be associated with the accumulation of active compounds in safflower stigmas.

### 3.5 Correlation network of safflower active components–soil properties–soil rhizosphere microorganisms

**Figure 5:**
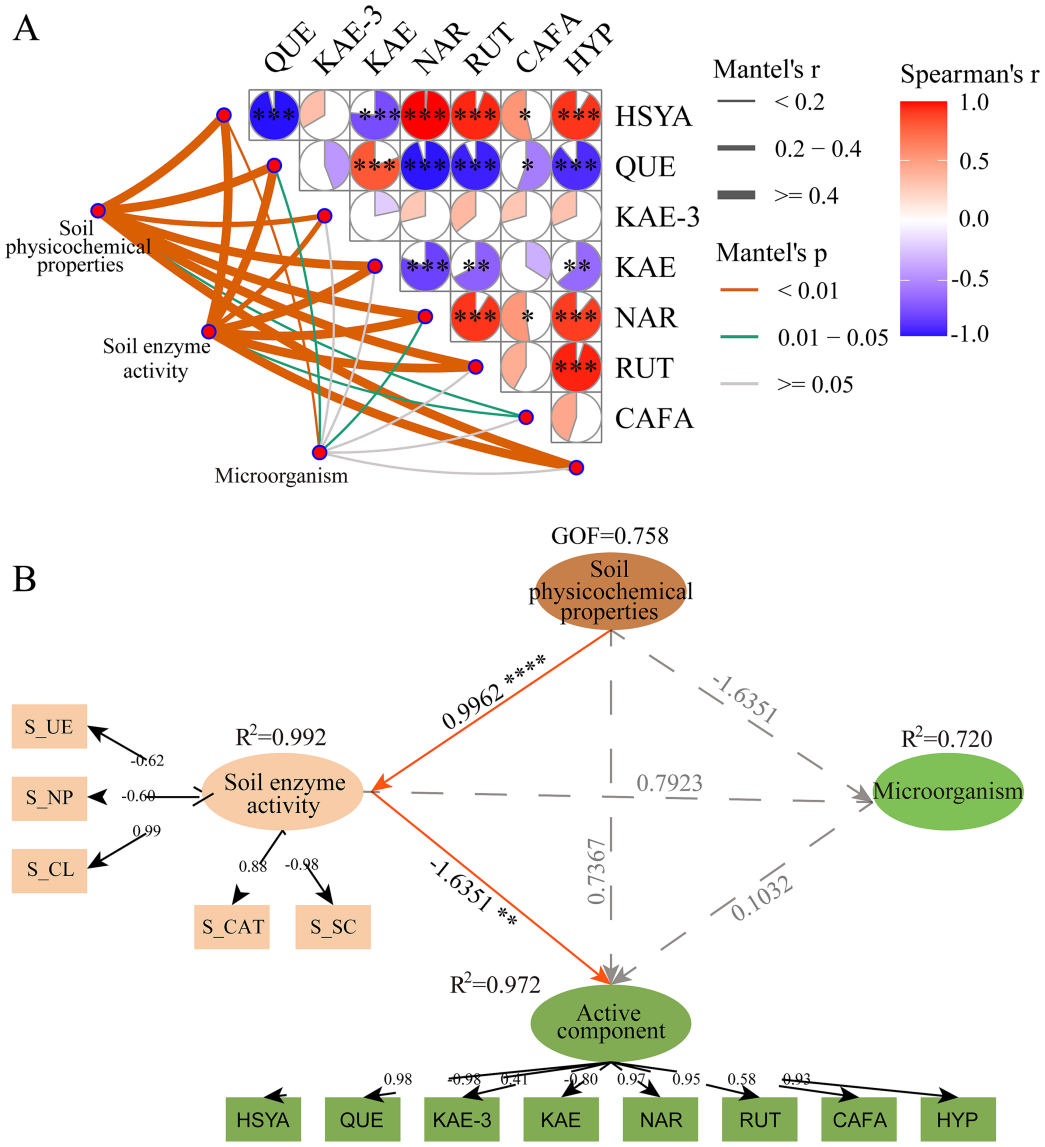
**(A)** Mental test correlation between safflower active components, soil physicochemical properties, and soil rhizosphere microorganisms. QUE, Quercetin; KAE-3, Kaempferol-3-O-rutinoside; KAE, Kaempferol; NAR, Naringenin; RUT, Rutin; CAFA, Caffeic acid; HYP, Hyperoside. **(B)** Partial east quares ath odel of afflower ctive omponents-oil hysicochemical roperties-oil hizosphere icroorganisms. Solid and dashed lines represent significant positive and non-significant pathways, respectively. Significance tests were conducted at *p* < 0.05. The model’s goodness-of-fit statistic was 0.758. Asterisks denote significant differences: * *p* ≤ 0.05; ** *p* ≤ 0.01; *** *p* ≤ 0.001; **** *p* ≤ 0.0001. Two-way ANOVA followed by Tukey’s HSD test. Data are presented as biological replicates for reproductive stage only (*n* = 9 independent samples per group, total *n* = 18).

Mantel tests and Spearman correlation coefficients analyzed the relationships between soil physicochemical properties, soil enzymes, soil rhizosphere microorganisms, and the primary active components of safflower stigmas (Figure 5A). Soil physicochemical properties and enzymes in the safflower rhizosphere showed significant positive correlations with microorganisms, HSYA, and caffeic acid. Soil rhizosphere microorganisms exhibited significant positive correlations with caffeic acid and HSYA. Rhizosphere soil physicochemical properties, enzymes, and microorganisms showed significant negative correlations with quercetin, kaempferol-3-O-rutinoside, kaempferol, naringenin, and rutin. Mental test analysis indicated correlations between rhizosphere soil physicochemical properties, enzymes, and microorganisms with the accumulation of active components in safflower stigmas. Among these, rhizosphere soil physicochemical properties showed the strongest correlation with microorganisms, HSYA, and caffeic acid; rhizosphere soil enzymes correlated most strongly with microorganisms, caffeic acid, and HSYA; while rhizosphere soil microorganisms showed the strongest correlations with caffeic acid, kaempferol-3-O-rutinoside, and HSYA.

Partial Least Squares Path Modeling (PLS-PM) indicated that the model adequately fitted the data, with an overall Goodness of Fit (GoF) value of 0.758 (Figure 5B). Soil physicochemical properties exerted a significant direct positive effect on the active components of safflower stigmas (path coefficient = 0.7367), indicating that soil characteristics significantly influence the accumulation of active components in safflower stigmas. A highly strong direct positive correlation existed between soil physicochemical properties and soil enzyme activity (path coefficient = 0.9962, *p* < 0.0001). Soil rhizosphere microbial communities showed a significant positive correlation with the active components in safflower stigmas (path coefficient = 0.1032). However, this effect was relatively weak and its mechanism remains unclear, suggesting that microorganisms may influence the accumulation of active components through indirect pathways such as regulating soil nutrient transformation, rather than through direct action. Soil physicochemical properties exerted a significant indirect effect on rhizosphere microbial communities by influencing soil enzyme activity (indirect effect value = 0.7923), indicating that soil enzyme activity plays a crucial mediating role between soil physicochemical properties and rhizosphere microbes. Furthermore, soil physicochemical properties indirectly influenced safflower stigmatic active components by affecting the rhizosphere microbial community. In summary, correlation analysis indicates that soil physicochemical properties, enzyme activity, and rhizosphere microbial communities are synergistically correlated with the active components of safflower, suggesting the potential existence of both direct and indirect associative pathways.

## 4 Discussion

This study correlates soil physicochemical properties, enzyme activity, and rhizosphere microbial communities with the content of active components in safflower stigmas. Results from two altitudinal regions indicate that AP, AN, and OM showed significant positive correlations with active ingredient content. Microbial community structures, including Actinobacteria, Proteobacteria, and Ascomycota phyla, also exhibited synergistic associations with HSYA accumulation. These findings provide correlational evidence for exploring soil-microbe-active ingredient relationships.

### 4.1 Influence of soil physicochemical properties on the accumulation of active components in safflower stigmas

This study identified significant correlation patterns between the accumulation of active components in safflower from two distinct production areas and soil fertility indicators. A key finding is that soil total phosphorus (TP) and available phosphorus (AP) levels in YM soil were significantly higher than in YF soil (Figure 3A), and this trend positively correlated with hydroxystigmatic acid (HSYA) content (Figure 3B). This represents a novel phosphorus-related correlation characteristic reported in safflower in this study. Previous literature indicates that the growth of understory medicinal herbs like turmeric significantly depends on the supply of available phosphorus and readily available potassium in the soil (Behera et al., 2022). However, lithospermum exhibits the highest active ingredient content in soils rich in readily available potassium. In contrast, safflower exhibits stronger phosphorus dependency, potentially linked to the reliance of flavonoid synthesis on the pentose phosphate pathway (Yao et al., 2024), though this requires metabolomics validation. Another finding concerns altitudinal variations in soil enzyme activity: S-NP enzyme activity in the YM plot increased synchronously with organic matter content, yet pH was significantly lower than in YF (Figure 3A). This aligns with previous reports indicating that lithospermum exhibits highest active ingredient content in low-pH, organic-rich soils. During the transition from the vegetative rooting (VR) to the root establishment (RR) stage, pH significantly increased (Figure 3A). In the VR stage, rapid root expansion and vigorous respiration may have caused rhizosphere acidification due to an imbalance between organic acid secretion and ion uptake. In contrast, during the RR stage, reduced root activity and decreased organic acid secretion led to a marked pH increase. This pH dynamic directly influences nutrient uptake (Rosell et al., 2023), though this hypothesis requires validation through rhizosphere secretion analysis. In summary, this study preliminarily confirms significant correlations between soil AP, AN, OM, and pH with nutrient requirements and metabolite levels at different growth stages of safflower, providing a basis for precision fertilization and soil management. However, the universality of these findings requires validation across broader geographic ranges and larger sample sizes.

### 4.2 Correlation characteristics between soil rhizosphere microbial communities and active components of safflower

This study revealed distinct differences in the rhizosphere microbial community structure between safflower origins YM and YF. These variations, together with soil nutrients and active component content, form an interconnected network. However, the causal relationship between microorganisms and active component accumulation remains unclear, with their effects likely being indirect.

Regarding bacteria, the phylum Actinobacteria dominated in both origins (Figure 4C). Specifically, the genera Rubrobacter and Arthrobacter were identified as core bacterial genera (Figure 4E). Literature suggests these genera may maintain soil nutrient cycling and microbial stability by regulating the rhizosphere microenvironment, enhancing nutrient uptake, and suppressing pathogens (Hu et al., 2020; Karthikeyan et al., 2018). Although literature reports suggest Arthrobacter may possess phosphorus solubilization capabilities (Zhang et al., 2020) and Rubrobacter is hypothesized to degrade complex organic matter (Kong et al., 2018), this study did not obtain direct evidence proving their direct promotion of HSYA synthesis. They are more likely to indirectly influence secondary metabolism by participating in rhizosphere phosphorus and carbon cycles, thereby improving plant nutritional status. The concurrent enrichment of phyla such as Gemmatimonadetes and Thaumarchaeota at the YM site further supports the potential involvement of rhizosphere microorganisms in phosphorus and nitrogen transformation (Li et al., 2024; You et al., 2024; Zhang R. Y. et al., 2024).

Regarding fungi, the Ascomycota phylum was identified as the dominant fungal phylum (Figure 4D). A key finding was the significant increase in relative abundance of the genus Gibberella across growth stages, with values significantly higher during the RR stage compared to the VR stage. This genus exhibits dual functions, potentially acting as both a pathogen (Wagacha and Muthomi, 2007; Zhang et al., 2023) and a producer of gibberellin (Aktar et al., 2023; Medeiros Araújo et al., 2020). However, in this study, its abundance showed weak correlation with active compound accumulation, leaving its specific ecological role in safflower unclear. Secondly, the ecological balance of fungal community structure is noteworthy. In the YM plot, the proportion of genera with biocontrol potential, such as Metarhizium and Chaetomium, increased with growth stage (Figure 4F) (Medeiros Araújo et al., 2020). In contrast, the YF plot exhibited enrichment of potential pathogens like Alternaria (Colque-Little et al., 2023; Viriyasuthee et al., 2019). Given the weak association between these fungal genera and active compounds, it is speculated that these microbial communities may indirectly influence final active compound accumulation by affecting safflower plant health rather than directly regulating metabolic pathways.

### 4.3 Effects of soil physicochemical properties-soil enzymes-soil rhizosphere microbial correlated network on the accumulation of active components in safflower stigmas

The study constructed a multifactorial correlation model demonstrating the synergistic influence of soil physicochemical properties, enzyme activity, and rhizosphere microorganisms on the accumulation of active components in safflower. The significantly higher soil available phosphorus (AP) and organic matter (OM) in the high-altitude production area (YM) provided a solid foundation for growth and development. This was achieved by enhancing key enzyme activities such as S-NP and S-UE, thereby driving nutrient mineralization (Hashidoko, 2005; Tian et al., 2019). Additionally, a soil nutrient-microbe synergy model exists: core microbial communities enriched in YM, such as the Arthrobacter genus, may form a “highly efficient rhizosphere nutrient transformation system” by activating phosphorus and degrading organic matter (Zhang et al., 2020). However, this inference is based on correlation analysis and requires verification through sterile inoculation experiments. Among the interactions between soil physicochemical properties, soil enzymes, and rhizosphere microbial communities, soil physicochemical factors show a more direct correlation with the accumulation of primary active components in safflower stigmas, while microbial factors introduce additional regulatory complexity.

## 5 Conclusion

This study employed high-throughput sequencing technology to systematically analyze the soil-microbe-active ingredient interaction networks across two safflower production sites at differing elevations. Results indicate that soil available phosphorus (AP), available nitrogen (AN), and organic matter (OM) significantly correlate with active ingredient content, particularly hydroxy-styrene-y-acetarbutin (HSYA). In contrast, rhizosphere microbial community structure—dominated by Actinobacteria and Proteobacteria among bacteria, and Ascomycota among fungi—showed weaker associations with active ingredient content. Microbial group differentiation at different altitudes and growth stages showed significant correlations with soil properties, though their associations with microenvironments and physiological states require further validation. This study provides insights into the soil ecological mechanisms underlying safflower quality formation and offers a reference framework for studies on authentic origin.

## Data Availability

The raw data supporting the conclusions of this article will be made available by the authors, without undue reservation.
